# PPARs and Their Neuroprotective Effects in Parkinson’s Disease: A Novel Therapeutic Approach in α-Synucleinopathy?

**DOI:** 10.3390/ijms24043264

**Published:** 2023-02-07

**Authors:** Isaac Pérez-Segura, Alberto Santiago-Balmaseda, Luis Daniel Rodríguez-Hernández, Adriana Morales-Martínez, Hilda Angélica Martínez-Becerril, Paola A. Martínez-Gómez, Karen M. Delgado-Minjares, Citlaltepetl Salinas-Lara, Irma A. Martínez-Dávila, Magdalena Guerra-Crespo, Francisca Pérez-Severiano, Luis O. Soto-Rojas

**Affiliations:** 1 Laboratorio de Patogénesis Molecular, Laboratorio 4, Edificio A4, Carrera Médico Cirujano, Facultad de Estudios Superiores Iztacala, Universidad Nacional Autónoma de México, Tlalnepantla 54090, Mexico; 2 Red MEDICI, Carrera Médico Cirujano, Facultad de Estudios Superiores Iztacala, Universidad Nacional Autónoma de México, Tlalnepantla 54090, Mexico; 3 Laboratorio de Medicina Regenerativa, Facultad de Medicina, Departamento de Fisiología, Universidad Nacional Autónoma de México, Mexico City 04360, Mexico; 4 Sección de Estudios de Posgrado e Investigación, Escuela Superior de Medicina, Instituto Politécnico Nacional, Mexico City 11340, Mexico; 5 Departamento de Fisiología, Biofísica y Neurociencias, Centro de Investigación y de Estudios Avanzados del IPN, Mexico City 07360, Mexico; 6 Departamento de Neuropatología, Instituto Nacional de Neurología y Neurocirugía Manuel Velasco Suárez, Mexico City 14269, Mexico; 7 Laboratorio de Neurofarmacología Molecular y Nanotecnología, Instituto Nacional de Neurología y Neurocirugía Manuel Velasco Suárez, Mexico City 14269, Mexico

**Keywords:** α-synucleinopathy, neuroprotection, Parkinson’s disease, Lewy bodies, PPAR, glitazones

## Abstract

Parkinson’s disease (PD) is the most common α-synucleinopathy worldwide. The pathognomonic hallmark of PD is the misfolding and propagation of the α-synuclein (α-syn) protein, observed in post-mortem histopathology. It has been hypothesized that α-synucleinopathy triggers oxidative stress, mitochondrial dysfunction, neuroinflammation, and synaptic dysfunction, leading to neurodegeneration. To this date, there are no disease-modifying drugs that generate neuroprotection against these neuropathological events and especially against α-synucleinopathy. Growing evidence suggests that peroxisome proliferator-activated receptor (PPAR) agonists confer neuroprotective effects in PD, however, whether they also confer an anti-α-synucleinopathy effect is unknown. Here we analyze the reported therapeutic effects of PPARs, specifically the gamma isoform (PPARγ), in preclinical PD animal models and clinical trials for PD, and we suggest possible anti-α-synucleinopathy mechanisms acting downstream from these receptors. Elucidating the neuroprotective mechanisms of PPARs through preclinical models that mimic PD as closely as possible will facilitate the execution of better clinical trials for disease-modifying drugs in PD.

## 1. Introduction

**Figure 1:**
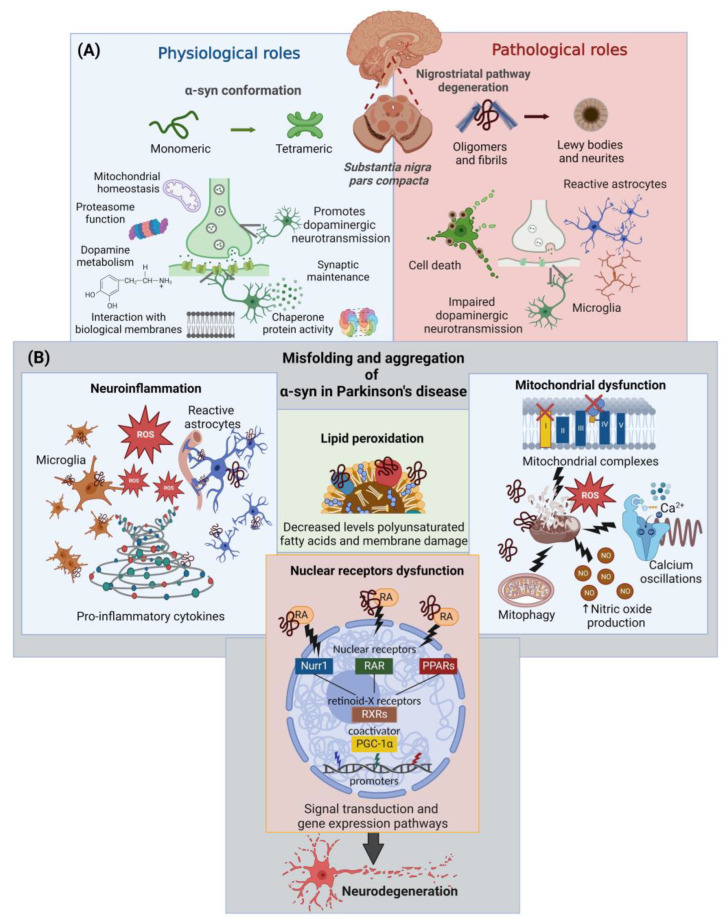
Physiological and pathological roles of α-syn in PD. (**A**) Physiological and pathological roles of α-syn according to its structural conformation in dopaminergic neurons of the *substantia nigra pars compacta* and their projections to the striatum. (**B**) Cellular alterations triggered by α-syn misfolding. Abbreviations: α-syn: α-synuclein; NO, nitric oxide; RA, retinoic acid; Nurr1, nuclear receptor 1; RAR, retinoic acid receptors; PPARs, peroxisome proliferator-activated receptors; RXR, retinoid X receptor; PGC1α, peroxisome proliferator-activated receptor gamma-1 α coactivator; ROS, reactive oxygen species. This figure was created with BioRender.com (accessed on 3 October 2022).

The term “α-synucleinopathy” is used to include a group of neurodegenerative diseases characterized by the pathological aggregation of α-synuclein (α-syn) in neuronal or glial cells and prion-like spreading through neuroanatomically interconnected regions [1]. These diseases include Parkinson’s disease (PD), which is the most common condition, dementia with Lewy Bodies (DLB), multiple system atrophy (MSA), as well as other rare disorders and neuroaxonal dystrophies [2,3]. PD is a chronic neurodegenerative disorder that represents a major cause of disability, with an estimated worldwide prevalence of 6.2 million affected, which is expected to double by 2040 due to risk factors such as increased life expectancy and exposure to environmental toxins [4,5]. Clinically, the PD patient presents motor symptoms such as resting tremor, bradykinesia, rigidity, and postural instability, as well as non-motor disturbances such as hyposmia, gastrointestinal and sleep dysfunctions, depression, and anxiety, among others, even decades before the motor symptoms first appear [6,7]. Histopathologically, PD is characterized by progressive loss of dopaminergic neurons in the *substantia nigra pars compacta* (SNpc), causing a deficiency in dopaminergic transmission in the nigrostriatal pathway [7]. In addition, the histopathological hallmark of PD is the presence of Lewy bodies, cytoplasmic inclusions composed predominantly of misfolded α-syn in both, genetic and sporadic etiologies [8,9]. The α-syn is a small protein 140 amino acids long, which can adopt different conformations depending on the environment and easily interacts with other ligands such as lipids. This protein is physiologically expressed at high concentrations in the brain and is involved in many critical neurological processes such as [2,10,11]: synaptic maintenance, mitochondrial and plasmatic membrane homeostasis, proteasome function, dopamine metabolism, and chaperone protein activity (Figure 1A).

The toxicity of this protein is related to structural modifications ranging from its oligomerization to aggregation in fibrils and, finally, its deposition in Lewy bodies and neurites (Figure 1A). The misfolded α-syn has been associated with several neuropathological events culminating in neurodegeneration (Figure 1B) [11,12,13,14,15,16]: (i) mitochondrial dysfunction; (ii) increased levels of oxidative stress, caused by high levels of reactive oxygen species (ROS) and lipid peroxidation (LP); (iii) decreased levels of polyunsaturated fatty acids and membrane damage; (iv) neuroinflammation; (v) alterations in calcium homeostasis; and (vi) nuclear receptor dysfunction.

Currently, there is no disease-modifying therapy against α-synucleinopathy. However, peroxisome proliferator-activated receptor (PPAR) agonists are excellent candidates that have demonstrated neuroprotective effects in preclinical PD models, specifically the gamma isoform (PPARγ). PPAR activity reduces oxidative stress, improves mitochondrial function, and reduces neuroinflammation and neurodegeneration of dopaminergic neurons in the SNpc and other brain areas [17]. This review critically presents the evidence of the neuroprotective effects of PPARγ in preclinical PD models and patients, and proposes potential anti-α-synucleinopathy effects of PPARγ agonists that may become suitable disease-modifying drugs.

## 2. Current Treatment of Parkinson’s Disease

Despite its high prevalence worldwide, conventional treatment of PD remains symptomatic, using pharmacological, non-pharmacological, and surgical treatments that seek to maximize the remaining motor and non-motor functions, as well as the quality of life [18,19]. Since its introduction more than 60 years ago, the replacement of striatal dopamine loss through systemic administration of the dopamine precursor amino acid (levodopa) has been a mainstay of treatment [7,20] because it shows the greatest positive effect on motor symptoms and quality of life [21].

Controversy exists about when to initiate pharmacological PD treatment [22]. Clinical trials found no evidence that early initiation of therapy has disease-modifying effects or a difference in the frequency of complications with late-onset [23,24]. Therefore, the initiation of treatment considers the characteristics of the severity of the disease in each patient [7,8]. However, long-term treatment (5–10 years) with levodopa and other dopamine agonists is associated with motor side effects in up to 10–40% of patients and, in some cases, with the appearance of non-motor effects [25]. Among the drugs available, levodopa requires the highest daily dosage and therefore carries a greater risk of side effects [26]. Its presentation in formulations, together with the decarboxylase inhibitor, carbidopa, prevents these effects by avoiding the peripheral conversion of levodopa [20]. Moreover, the use of formulations that include catechol-O-methyltransferase inhibitors decreases the risk of motor fluctuations by increasing their plasma half-life through pharmacokinetic modifications [20,26].

Dopamine agonists (apomorphine, pramipexole, ropinirole) and monoamine oxidase inhibitors (selegiline, rasagiline) have also been used as initial PD treatment [18], but with poorer tolerability and less improvement in mobility scores, with no evidence of delaying motor fluctuations [7,8]. Therefore, their greatest usefulness lies as an adjuvant therapy when an enhanced treatment effect is needed without increasing the doses of levodopa in the advanced stages of the disease [18,26]. For those patients with motor fluctuations that do not respond to medication adjustments, alternative therapies are used to achieve continuous dopaminergic stimulation, such as deep brain stimulation, magnetic resonance imaging-guided focused ultrasound, and levodopa-carbidopa intestinal gel pump therapy. However, these alternative therapies require evaluations at specialized centers to determine patient eligibility, ongoing medication management, and device optimization [7,8].

To sum up, there is no disease-modifying drug so far [7,27], however, the side effects of conventional antiparkinsonian treatment and the complexity of alternative (advanced) therapies have driven the search for new therapeutic targets [8,27,28,29] with the main objectives of preventing neurodegeneration [20], reducing oxidative stress while stimulating neurotrophic factors [30,31], supporting calcium homeostasis and lysosomal autophagy preventing mitochondrial dysfunction [32], controlling α- syn fibrils [33], promoting neuronal regeneration [34] and employing pharmacoepigenomics based therapies [35]. As discussed in the following sections, the activation of certain nuclear receptors, such as the PPARs, could be useful in PD treatment since they have been observed to exert a positive effect on several of these therapeutic targets.

The modifications of α-syn play a central role in PD pathology while triggering multiple neuropathological events that culminate in neurodegeneration. The above could be due to disrupted interactions between α-syn and diverse nuclear receptors [36]. Therefore, pharmacological modulation of specific nuclear receptors, such as the PPARs, could be helpful in PD treatment since they have been observed to provide neuroprotective effects in various central nervous system (CNS) diseases [17] and since their use for the treatment of other pathologies has shown a collateral reduction of PD incidence [37,38].

## 3. Pathological Impact of α-Syn on Nuclear Receptors in Parkinson’s Disease

The α-syn aggregates have been associated with alterations at a nuclear level, particularly affecting transcriptional regulation [39,40]. Oxidative stress and mitochondrial dysfunction promote the translocation of α-syn to the nucleus, where the specific function of α-syn remains uncertain [41,42]. Also, several α-syn mutations in the human population, such as *A30P*, *A53T*, and *G51D*, promote their accumulation in the nucleus [43,44]. In this context, α-syn aggregates have been described to modify the activity of nuclear receptors associated with the gene expression of dopaminergic neurons [45]. One of them is nuclear receptor 1 (Nurr-1), a member of the inducible orphan nuclear receptor family. This receptor is essential in the differentiation, maturation, and survival of midbrain dopaminergic neurons [46], which have been reported to be predominantly affected by α-syn aggregation in PD [47]. In addition, the pathological α-syn negatively regulates the transcriptional activity of Nurr-1, and the receptors that modulate the transcription of retinoic acid receptors (RARs), and peroxisome proliferator-activated receptor gamma-1α coactivator (PGC-1α) [45,47,48]. The latter is a transcriptional regulator involved in mitochondrial biogenesis and cellular energy metabolism that are affected in PD [48,49]. RARs promote PPAR activity, which is also affected in PD. The latter two types of receptors require heterodimerization with retinoid X receptors (RXRs) to form heterodimers capable of exerting transcriptional functions on specific target genes [45].

Importantly, nuclear translocation of α-syn has been shown to decrease PPARγ activity, compromising cell survival (Figure 1B) [45,50]. Since α-synucleinopathy is associated with different neuropathological mechanisms, including dysfunction of nuclear factors involved in the survival and maintenance of dopamine neurons, current therapy approaches should confer neuroprotective effects by preventing or decreasing the α-syn misfolding.

## 4. PPARs: Types, Distribution and Functions

PPARs belong to subgroup 1 of the nuclear receptor superfamily [51]. They are known to form heterodimers with the RXR when activated by endogenous or exogenous ligands and to bind to a co-activator such as PGC-1α. The activated PPAR complex binds to peroxisomal proliferative-response elements (PPREs), promoting gene transcription. Three isoforms of these receptors are known as the α, γ, and β/δ isoforms. However, the *PPARγ* gene generates three transcripts by alternative splicing encoding for further γ isoforms [52], which are involved in lipid metabolism, mitochondrial biogenesis, cellular energy production, glucose and amino acid regulation, and thermogenesis [53]. Also, PPARs are activated by lipids consumed in the diet (fatty acids) or their metabolites (such as eicosanoids), and they are considered to be lipid sensors [54].

PPARs are ubiquitously expressed in the organism, as shown in Table 1, while the β/δ isoform is mainly expressed in the CNS, however, the γ isoform is the most studied therapeutic target in several neurodegenerative diseases [55]. According to data retrieved from the Genotype-Tissue Expression (GTEx) project, the region with the most abundant expression is the caudate nucleus for α isoform, the cerebellar hemisphere for γ, and the cerebellum for β/δ. In addition, several nuclei from the basal ganglia are included, including the same ones that play an essential role in the motor deterioration of diseases such as PD and Huntington’s disease (HD). On the other hand, Table 1 highlights the agonists corresponding to each isoform. It is worth mentioning that fatty acids and their derivatives could activate all isoforms and are considered pan-agonists or/and endogenous agonists. According to some research groups, there is a connection between the three isoforms called “the PPARs triad”. Activation of the triad regulates neuroprotection by promoting PPAR-dependent genes, including positive feedback on PPARs themselves [56]. PPARγ increases the levels of the β/δ isoform, and vice versa, PPARβ/δ increases PPARγ levels. In addition, the β/δ isoform regulates α and γ activation, inducing the production of their endogenous agonists [57]. According to the PPAR triad theory, PPARγ is essential for triad maintenance even in regions of the CNS where the abundance of the β/δ isoform predominates.

Table 1 shows the ligands for PPAR divided into endogenous and exogenous, but these ligands can also act as selective agonists for one isoform, agonists with dual effect, or pan-agonists. The neuroprotection that agonists can provide depending on their mode of action is discussed in what follows.

Several animal models of PD, HD, and Alzheimer’s disease (AD) have shown a neuroprotective effect of PPARγ activation by agonists [55,58,59]. Glitazones (rosiglitazone, pioglitazone, and lobeglitazone) are the most widely studied PPARγ ligands, indicating the importance of this isoform [60]. The main effects of specific PPARγ activation include prevention of mitochondrial dysfunction, reduction of ROS, LP production, increased PGC-1α production, suppression of autophagy, maintenance of mitochondrial membrane potential (ΔΨm), inhibition of the proinflammatory cytokines, preservation of dopaminergic neurons, and reduction of macrophage infiltration [61,62,63,64,65].

On the other hand, recent studies point to the PPARα isoform being the target for preventing damage in AD, PD, depression, and schizophrenia [66,67,68]. Fibrates (fenofibrate, clofibrate) are the main agonists of the α isoform, and recent studies have shown a neuroprotective effect, especially in the case of gemfibrozil [69,70]. The different neuroprotective mechanisms related to PPARα activation are: (a) maintenance of glutamate homeostasis; (b) regulation in the metabolism of amyloid beta (Aβ) peptide; (c) cholinergic/dopaminergic signaling in the CNS; (d) attenuation of behavioral changes and dopaminergic dysfunction; (e) antidepressant activity; and (f) decreased proinflammatory signals and astrogliosis [66,67,71,72].

Regarding the β/δ isoform, some specific agonists known are L-165041, GW0742, and KD3010; the last one reported as safe in the Phase 1b Clinical Trial for metabolic disorders treatment, including obesity [73,74,75]. Activation of the β/δ PPAR isoform resulted in neuronal protection in various brain pathologies, such as cerebral ischemia, multiple sclerosis, amyotrophic lateral sclerosis, HD, PD, and AD [74,76,77,78,79,80,81]. In addition, neuroprotective effects conferred by PPAR β/δ activation include the regulation of ceramide metabolism, the reduction of (Aβ) aggregates, anti-inflammatory and antiapoptotic activity, prevention in mitochondrial dysfunction, decreased neutrophil infiltration, diminished oxidative stress and synthesis of antioxidant enzymes, ultimately leading to the restoration of cognitive functions [74,75,77,78,82].

Some ligands also exhibit dual effects, for example, 4-hydroxynonenal (4-HNE)-mediated PPAR β/δ antagonist/PPAR γ agonist has been verified to counteract the primary and secondary signs of PD neurodegeneration [83]. In addition, MHY908, a PPAR α/γ dual agonist, prevents the loss of dopaminergic neurons and motor deficits in a PD model [84].

Finally, the known endogenous agonists are considered to be pan-agonists. Belonging to these ligands are some fatty acids. However, we also find agonists of similar lipidic nature that are exogenous (consumed by the diet; see Table 1), as in the case of exogenous fatty acids (oleic acid, eicosapentaenoic acid, and docosahexaenoic acid) that promote PPARγ receptor expression [85]. Also, synthetic pan agonists (GFT1803 and bezafibrate) have been reported to prevent brain glucose hypometabolism and neuronal loss, attenuate microgliosis and the development of behavioral features in models of AD and Tau pathology [86,87].

**Table 1:** Expression of PPARs in the brain and their neuroprotective effects.

Receptor Isoform/Agonist	Brain Expression (TPM *)	Neuroprotection Effects	Ref.
**PPAR-α** *Endogenous*: Fatty acids such as palmitic, stearic, palmitoleic, oleic, linoleic, AA and EPA. *Exogenous*: WY-14643, clofibrate, gemfibrozil, nafenopin, bezafibrate, and fenofibrate.	Cd (4.799), Sc (4.660), SN (4.562), Acc (4.402), Acb (4.398), Cx (4.241) Amg (4.204), Pu (3.801), FroCx (3.677), Hy (3.597), Cb (3.561), HiF (3.057), CbH (2.399).	Participates in neurotransmission processes, decreases neuroinflammation, oxidative stress, and Aβ aggregation.	[59,88]
**PPAR-β/δ** *Endogenous*: EPA, linoleic acid, 13-S-HODE, and 4-HNE. *Exogenous:* WY-14643, GW0742, GW501516, KD3010, and L-165041.	Cb (46.72), CbH (42.24), Cx (36.76), FroCx (34.37), HiF(27.25), Sc (26.71), Acc (26.45), SN (22.51), Hy (21.20), Acb (20.36), Cd (20.27), Pu (18.38), Amg (2.77).	Prevents damage in neurodegeneration (AD, PD, HD, MS, and ALS) ischemia, CNS traumatic injury, and neuroinflammation.	[74,79,80,81,83,89]
**PPAR-γ** *Endogenous*: AA, EPA, and 15 deoxy PGJ12. *Exogenous:* Pioglitazone, Rosiglitazone Ibuprofen, piroxicam, ciglitazone, and GW1929.	CbH (2.744), Cb (2.425), FroCx (2.175), Acc (1.835), Cx (1.834), Acb (1.546), Sc (1.473), HiF (1.344), Amg (1.268), Hy (1.058), Cd (0.9625), SN (0.8271), Pu (0.7124).		

Abbreviations: Acb, nucleus accumbens; Acc, anterior cingulate cortex; AD, Alzheimer’s disease; ALS, Amyotrophic lateral sclerosis; Amg, amygdala; AA, arachidonic acid; Cb, cerebellum; Aβ, amyloid beta; CbH, Cerebellar Hemisphere; Cd, caudate nucleus; CNS, central nervous system; Cx, cortex; EPA, eicosapentaenoic acid; FroCx, frontal cortex; HD, Huntington’s disease; HiF, hippocampal formation; Hy, hypothalamus; MS, multiple sclerosis; PD, Parkinson’s disease; PG, pituitary gland; Pu, putamen; Sc, spinal cord (cervical c-1), SN, *substantia nigra*; TPM: transcripts per million; 15 deoxy PGJ12, 15 deoxy PGJ2, 15-Deoxy- ∆-12,14-Prostaglandin J2; 13-S-HODE, 13-S-hydroxyoctadecadienoic acid; 4-HNE, 4-hydroxynonenal; * Data Source: GTEx Analysis Release V8 (dbGaP Accession phs000424.v8.p2).

## 5. PPARγ in Preclinical Models of Parkinson’s Disease

Several preclinical trials have used PPARγ agonists in different PD animal models, attempting to demonstrate their positive counter-effects on ROS generation, mitochondrial dysfunction, neuroinflammation, neurogenesis, and the loss of dopaminergic neurons. It has been suggested that PPARγ agonists mediate the above processes through the production of paraoxonase-2 (PON2), an enzyme highly expressed in dopamine-rich brain regions, which enhances the function of coenzyme Q in the electron transport chain and, subsequently, reduces ROS production [90]. Table 2 summarizes the different neuroprotective effects of PPARγ agonists in preclinical PD models.

On the other hand, it is important to mention that some of these preclinical models that were used to evaluate the effects of PPAR agonists, have a disadvantage that they do not reproduce misfolded α-syn, while the acuity of dopaminergic neuronal death or neuroinflammation may also differ from the situations encountered in patients [91]. Therefore, it is essential to evaluate the proposed neuroprotective effects of PPAR agonists, including the anti-α-synucleinopathy effect, in a preclinical model capable of progressively reproducing the PD hallmarks. Interestingly, to this date, there have been no preclinical studies have been performed to evaluate the therapeutic effects of glitazones in invertebrates, or genetic models overexpressing α-syn.

Recently, our working group has developed an animal model of PD using a single intranigral administration of β-sitosterol β-D-glucoside (BSSG), which is a neurotoxin isolated from the plant *Cycas micronesica* belonging to the family of β-sitosterols. This animal model reproduces motor and non-motor alterations, induces the chronic, progressive, and irreversible death of dopaminergic neurons, neuroinflammation, and aggregation and propagation of misfolded α-syn [92,93]. Since this model reproduces most of the pathognomonic features of PD, it can be used to evaluate the neuroprotective and anti-α-synucleinopathy effects of PPARs agonists.

**Table 2:** Preclinical trials with different PPARγ agonists in several animal models of Parkinson’s disease.

Animal Model	Dosage and Administration Route	Neuroprotector Effect	Ref.
**MPTP Intranigral** Male Wistar rats.	MPTP + Pioglitazone 10 mg/kg PO for 5 days before MPTP injection and 30 days thereafter.	Lowering malondialdehyde and increasing glutathione levels.	[94]
**Intranigral 6–OHDA** Male Wistar rats.	Pioglitazone 30/mg/kg PO every 24 h for 5 days.	Decreased microglia activation and NF-kB expression.	[95]
**Bilateral intranigral 6-OHDA** Male Wistar rats.	1. Pioglitazone 30 mg/kg PO every 24 h for 5 days before administration of 6-OHDA; 2. Pioglitazone 30 mg/kg PO every 24 h for 5 days before 6-OHDA.	Reduction of neuronal death and microglial activation in SN. Increased antidepressant effects and the neuron’s survival and neurogenesis in the hippocampus.	[96]
**MPTP intranigral** Male mice C57/B16.	Pioglitazone 20 mg/kg/day PO in rodent food 4 days before MPTP administration.	Increased DA levels in the striatum and decreased microglial activity and iNOS expression.	[97]
**Bilateral MPTP intranigral** Male Wistar rats.	Pioglitazone 30 mg/kg or PO every 24 h for 5 days before MPTP administration.	Protects against dopaminergic neurodegeneration.	[70]
**6-OHDA intranigral** Male Sprague-Dawley rats.	Pioglitazone (20 mg/kg), GW855266X (Partial PPARγ agonist; 15 mg/kg), were administrated 7 days before and 7 days after 6 OHDA.	Pioglitazone protected against dopaminergic neuron loss in the SN, striatal dopamine depletion, and microglia activation.	[98]
**Subcutaneous rotenone** Male Wistar rats.	Pioglitazone 10 mg/kg IP at the end of rotenone administration + retinoic acid 1 mg/kg IP for 15 days.	Both agonists reversed the locomotor alteration. Pioglitazone increased the level of striatal dopamine.	[99]
**MPTP/probenecid (MPTPp)** C57BL/6J mice.	Rosiglitazone 10 mg/kg was given daily until sacrifice, starting on the fourth week of MPTPp treatment.	Rosiglitazone reverted microglial activation, TNF-α expression, and the nigrostriatal degenerative process.	[64]
**6-OHDA** Male Sprague-Dawley rats.	Rosiglitazone 3 mg/kg IP at 24 h and 30 min before lesion.	Prevented dopaminergic neuron loss and microglial activation in the striatum.	[100]
**MPTP IP** (3–5 doses every 24 h) Male C57B1/6J mice.	Rosiglitazone 10 mg/kg IP administered daily, 1 h before MPTP and until death (5 weeks of treatment).	Prevented dopaminergic neuron loss in the SN, and dopamine loss in caudate-putamen (partially). Inhibited microglia reactivity in SN and caudate-putamen, and partially astroglial response.	[101]
**Rotenone, streptozocin, and a high-calorie diet.** Male Wistar rats.	Lobeglitazone 0.1, 0.2, or 1.0 mg/kg IP one hour before rotenone injections for 46 days.	Prevented decrease of TH and the increase of TNF-α and NF-κB levels in SN and striatum.	[102]

Abbreviations: 6-OHDA, 6- Hydroxydopamine; DA, dopamine; iNOS, inducible nitric oxide synthase; IP, intraperitoneal; MPTP, 1-methyl-4-phenyl-1,2,3,6-tetrahydropyridine; NF-kB, nuclear factor kappa-light-chain-enhancer of activated B cells; PO, per os; PON2, Paraoxonase-2; PPAR, peroxisome proliferator-activated receptors; SN, substantia nigra; TH, tyrosine hydroxylase; TNF-α, tumoral necrosis factor-α.

## 6. Clinical Trials in Parkinson’s Disease Using PPARγ Agonists

We searched the ClinicalTrials.gov website and found only one randomized, multicenter, double-blind, placebo-controlled, futility clinical trial organized by the Clinical Trials Coordination Center (CTCC) at the University of Rochester, NY, USA, which tested the use of pioglitazone in early PD [73]. In this study, participants were assigned to three different groups: (1) 15 mg/day pioglitazone; (2) 45 mg/day pioglitazone; and (3) placebo for 44 weeks. They evaluated changes in different PD progression scales (Unified Parkinson Disease Rating Scale), showing no significant improvement over the use of pioglitazone as a disease-modifying drug for PD [103]. However, there were some limitations in this study. First, the use of pioglitazone together with rasagiline might have biased the results. Second, the therapeutic effect of this PPARγ agonist was only evaluated for a short period (44 weeks), and therefore, in relation to the results obtained, more time could be necessary to show significant effects, considering the potentially long prodromal PD period [8]. Third, the study did not use serological or cerebrospinal fluid biomarkers or imaging tests to assess the neuroprotective effects of pioglitazone. Fourth, another limitation of the study was that not all patients tolerated the drug doses; therefore, in general, the 44 weeks were concluded with different doses, depending on the tolerability of each patient. Finally, the odor identification test could not be performed as initially planned. In this context, from this single study, it is not possible to conclude the absence of beneficial effects of pioglitazone in PD.

Future clinical trials with PPARγ agonists, in the early stages of PD, are required to analyze their effect on clinical scales (both motor and non-motor manifestations), cabinet tests, as well as biomarkers that reflect neuroinflammation, oxidative stress, neurodegeneration, and the α-synucleinopathy. Therefore, the analysis of their efficacy in an animal PD model capable of reproducing most of the hallmarks of the human disease will be useful to determine the timing and neuroprotective mechanisms of glitazones.

## 7. Possible Anti-α-Synucleinopathy Role of Glitazones

**Figure 2:**
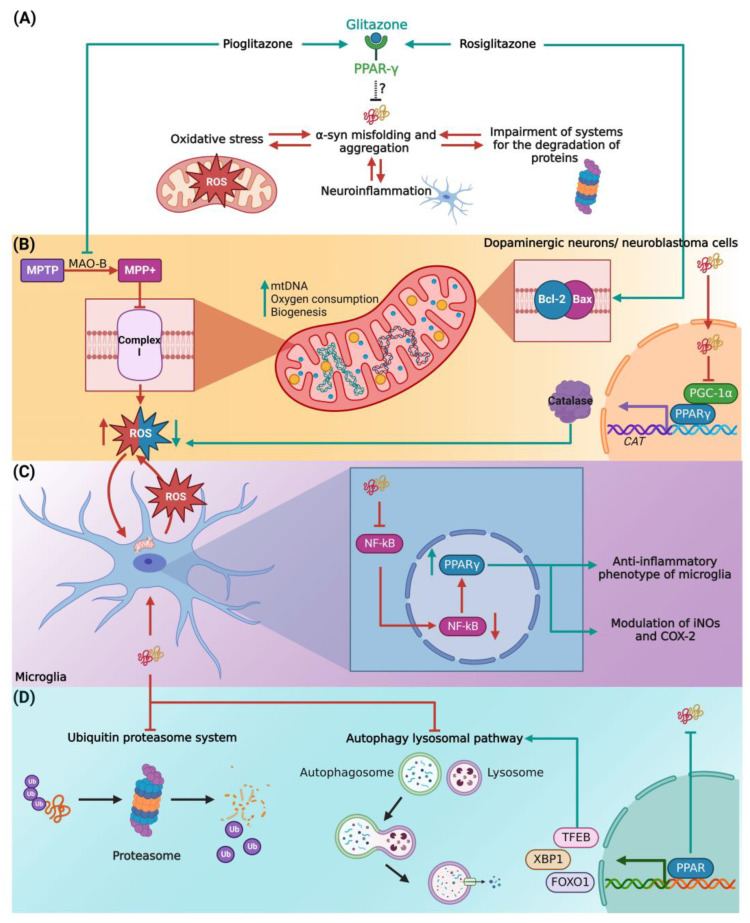
Proposed mechanisms of PPARs agonist α-synucleinopathy. (**A**) Glitazones as activators of PPARs may inhibit α-syn misfolding and aggregation in terms of reduction of (**B**) oxidative stress, (**C**) control of neuroinflammation, and (**D**) regulation of the protein degradation systems. Abbreviations: α- syn, α synuclein; COX-2, cyclooxygenase 2; FOXO1, Forkhead box protein O1; iNOS, inducible nitric oxide synthase; MAO-B, monoamine oxidase-B; MPP+, 1-methyl-4-phenylpyridinium; MPTP, 1-methyl-4-phenyl-1,2,3,6-tetrahydropyridine; mtDNA, mitochondrial DNA; NF-kB, nuclear factor kappa-light-chain-enhancer of activated B cells; PPAR, peroxisome proliferator-activated receptors; PGC1, peroxisome proliferator-activated receptor gamma-1 α coactivator; ROS, reactive oxygen species; TFEB, transcription factor EB; XBP1, X-box-binding protein 1. The figure was created with BioRender.com (accessed on 3 October 2022).

Evidence that PPARγ agonists have a neuroprotective effect in several animal PD models was presented above. However, despite being the central components in the neuropathology of PD, we were unable to identify any studies evaluating the impact of these agonists, on α-syn misfolding and Lewy body formation. This omission may be due to the limited availability of PD models representing the disease progression in terms of α-syn proteostasis loss. Nevertheless, the neuroprotective effect of glitazones could have a mechanistic basis directly related to decreasing, or preventing, α-syn aggregates (Figure 2A). To support this hypothesis, it is necessary to recapitulate the main neuropathological processes leading to the misfolding and aggregation of α-syn.

First, we considered the role of oxidative stress in α-syn misfolding and aggregation (Figure 2B). The high metabolic rate of dopaminergic neurons involves a large number of oxidative phosphorylation processes and the generation of ROS in mitochondria [104,105], which are capable of oxidizing proteins and destabilizing their tertiary conformation [106]. Indeed, the deficient activity of complex I of the respiratory chain is considered the main source of ROS in PD [107]. As such, 1-methyl-4-phenyl-1,2,3,6-tetrahydropyridine (MPTP), a complex I inhibitor, shows preferential cytotoxicity on dopaminergic neurons [108]. Therefore, the neuroprotection of the PPARγ agonist pioglitazone, by inhibiting the conversion of MPTP to the toxic metabolite 1-methyl-4-phenylpyridinium (MPP+), via inhibition of monoamine oxidase-B [109], could be useful to prevent or even reduce its effect on ROS formation in the mitochondria. In addition, pioglitazone has been associated with an increase in mitochondrial DNA content, expression of factors related to mitochondrial biogenesis, and transcriptional regulation of mitochondrial membrane transporters related to the control of energy metabolism in mitochondria [110,111]. Similarly, rosiglitazone has been shown to protect human neuroblastoma cells against acetaldehyde, another inhibitor of mitochondrial function, through the expression of antioxidant enzymes and upregulation of B-cell lymphoma protein 2 (Bcl-2) and Bcl-2 Associated X-protein (BAX) expression [112]. Collectively, the administration of PPARγ agonists could improve α-syn proteostasis and mitochondrial dysfunction by decreasing ROS production in PD. Also, it is important to mention that it is still unknown whether oxidative damage in PD results from excessive production or deficient clearance of oxidative agents [113,114,115]. In this regard, Yakunin et al. (2014) observed that aggregation of α-syn can directly affect the transcription of PPARγ [50], which regulates catalase transcription responsible for ROS scavenging. Consistent with this notion, nuclear α-syn binds to the PGC-1α promoter leading to the proposal that α-syn inhibits catalase activity through its effect on PGC-1α activity [116]. Thus, an external activation of this PPAR isoform by specific agonists could enhance α-syn-affected catalase activity and protect neurons from oxidative stress hindering, and in turn, the α-syn misfolding itself [116].

On the other hand, chronic neuroinflammatory responses and neuronal apoptosis play a significant role in the pathogenesis of PD, as evidenced by increased nuclear translocation, altered expression of nuclear factor kappa-light-chain-enhancer of activated B cells (NF-kB), p53 protein, and caspases in the SNpc [117]. Similarly, α-syn aggregation causes microglial activation leading to persistent and progressive nigral neurodegeneration in PD [118,119], primarily derived from an amplification of mitochondrial dysfunction, contributing to oxidative stress and the vicious cycle of α-syn aggregation [120]. In this line of argument, the three PPAR isoforms have been identified to regulate the transcriptional activity of several transcriptional factors (in addition to the aforementioned NF-κB) involved in the inflammatory response via modulation of inducible nitric oxide synthase (iNOS) and cyclooxygenase 2 (COX-2) [121]. Specifically, Li et al. (2020) identified a physiological response of microglia regulation toward an anti-inflammatory phenotype via PPARγ activation [122]. Thus, activation of PPARγ by specific agonists could result in a decrease of the chronic inflammatory response, leading to oxidative phenomena responsible for α-syn aggregation and cell death of dopaminergic neurons (Figure 2C).

Finally, another important factor in the pathophysiology of PD is the impairment of the systems responsible for the degradation of misfolded and aggregated α-syn, such as the ubiquitin-proteasome system [123] and the autophagy-lysosomal pathway [124,125]. Potentiation of the intracellular α-syn aggregate degradation systems could be a potential strategy against α-synucleinopathies. In this regard, it has been identified that PPARs stimulate the transcription of diverse transcription factors involved in lysosomal biogenesis and autophagy (Figure 2D), such as the transcription factor EB (TFEB), X-box-binding protein 1 (XBP1), and Forkhead box protein O1 (FOXO1) [126,127].

## 8. Effects of PPARs Agonists on Other Neurodegenerative Disorders

Nowadays, PPAR agonists are considered effective in various neurodegenerative diseases such as PD, AD, and HD. Interestingly, AD and PD share several pathological features, including increased incidence with age, chronic and progressive neuronal death, neuroinflammation, mitochondrial dysfunction from elevated ROS production, and protein misfolding [128,129,130]. However, specifically in AD brains, two pathognomonic features are observed mainly in the hippocampus [131,132]: (1) extracellular deposits of amyloid beta peptide (Aβ); and (2) intracellular aggregates of pathological tau protein. Recently, it has been postulated that endogenous “damage signals” such as Aβ oligomers or ROS, could cause microglial activation followed by the release of proinflammatory cytokines that trigger Tau hyperphosphorylation and aggregation, and when neurons die, Tau is released and causes microglial activation, generating a vicious circle that leads to neurodegeneration [133]. Unfortunately, most of the current therapeutic approaches have not been successful, since they focus on isolated, partial mechanisms of the overall pathology [134,135]. Therefore, the treatment of neurodegenerative diseases should be considered in light of the multiconvergent theory [136,137] in the face of various neuropathological events such as neuroinflammation, oxidative stress, and protein misfolding. In this context, PPAR analogs would be useful for the treatment of these neurodegenerative disorders [138,139,140,141,142]. For example, PPARγ agonists have been reported to modulate the expression of various AD-related genes, such as Bcl-2, which is involved in hippocampal neurodegeneration [143], and they have also been shown to reduce Aβ peptide levels both by increasing its clearance and by modifying the activity of secretases that are involved in its metabolism [144,145]. Likewise, it has been suggested that PPARs play a key role in the regulation of oxidative stress and neuroinflammation [140].

On the other hand, HD is a neurodegenerative disorder that affects movement and similar neuroanatomical structures as in PD. However, HD is characterized by the presence of involuntary choreatic movements, neuropsychiatric symptoms, and cognitive impairment [146,147]. It originates from a mutation in the huntingtin gene (HTT), which specifically causes aggregation of the huntingtin protein in the cortex and caudate/putamen [138]. Mitochondrial dysfunction is also related to the development of HD pathogenesis, and PPAR alteration plays an important role [76,148]. It has been found that overexpression of PGC-1α improves the motor phenotype, decreases neurodegeneration and the accumulation of the mutant huntingtin protein, by attenuating oxidative stress [149]. Likewise, activation of PPARδ and PPARγ by their agonists, KD3010 and rosiglitazone, respectively, increases survival, normalizes endoplasmic reticulum stress, reduces huntingtin aggregates, and improves mitochondrial function [76,150]. Therefore, these findings support the use of PPARs as a therapeutic strategy in neurodegenerative diseases.

Finally, a close association between neurodegenerative diseases and diabetes mellitus (DM) has been described [151,152]. DM constitutes a great global health challenge for around 460 million people worldwide [153]. According to preclinical assays, possible neuropathological mechanisms involved in these pathologies have been postulated [154,155]: (i) cerebrovascular disease; (ii) misfolding of proteins; (iii) chronic insulin resistance, which is associated with PGC-1α downregulation, mitochondrial complex I dysfunction, neuroinflammation, and impaired autophagy; (iv) amylin neuropathology. Amylin, a highly amyloidogenic pancreatic peptide, is increased in DM patients, can cross the BBB and accelerate α-syn [156] aggregation, Tau [157] phosphorylation, and Aβ [158] aggregates. Although DM patients treated with PPAR agonists have a lower risk of developing neurodegenerative disorders [159,160], evidence correlating PPAR dysfunction is lacking. Therefore, future preclinical and clinical trials are needed to evaluate the downregulation of PPARs in DM patients and their association with neurodegeneration.

## 9. Conclusions

Since there is currently no PD-modifying drug, treatment with PPAR agonists promises to establish more successful therapeutic strategies. PPAR agonists, in particular those activating the gamma isoform, have the property of being multiconvergent, acting through different targets, and providing multiple therapeutic effects: attenuation of the neuroinflammatory process, regulation of oxidative stress, promoting the synthesis of antioxidant mediators, and stabilizing mitochondrial function. In the present review, we hypothesized that these neuroprotective effects could converge in reducing, or preventing, the misfolding of α-syn and other proteins involved in other neurodegenerative disorders, and consequently promote clinical improvement. However, there is very limited scientific evidence on the use of PPARs agonists in PD, so more research is required using in vivo models that reflect the disease progression, especially elucidating the PPARs mechanisms on aggregation and misfolding of α-syn.

## Data Availability

No new data were created or analyzed in this study. Data sharing is not applicable to this article.
